# 2465: The design of a patient-centered personal health record with patients as co-designers

**DOI:** 10.1017/cts.2017.77

**Published:** 2018-05-10

**Authors:** Arlene Chung, Haiwei Chen, Grace Shin, Ketan Mane, Hye-Chung Kum

## Abstract

OBJECTIVES/SPECIFIC AIMS: The promise and potential of connected personal health records (PHRs) has not come to fruition. This may be, in part, due to the lack of user-centered design and of a patient-centric approach to curating personal health data for use by patients. Co-design with end-users could help mitigate these issues by ensuring the software meets user’s needs, and also engages patients in informatics research. Our team partnered with patients with multiple chronic conditions to co-design a patient-centric PHR. This abstract will describe our experience with the co-design process, highlight functionalities desired by patients, and showcase the final prototype. METHODS/STUDY POPULATION: We conducted 3 design sessions (90 min per session) with patients as co-designers and employed an iterative process for software development. Patients were recruited from Chapel Hill and surrounding areas. The initial design session laid the foundation for future sessions, and began with brainstorming about what patients thought their ideal version of an engaging connected PHR would look like in terms of features and functionalities. After each software iteration, our entire design team, including our patient co-designers, was shown the prototype during a subsequent design session. Once the final prototype was developed, usability testing was conducted with patient participants. Our team then conducted a final design session to debrief about the final prototype. RESULTS/ANTICIPATED RESULTS: We started with an initial group of 12 patients (6 males) who all had diabetes and an additional comorbidity such as hypertension and hyperlipidemia. Age of participants ranged from 30 to 77 years with an average age of 56. The majority of participants were Caucasian with 1 Asian and 2 African Americans. Hemoglobin A1c values ranged from 6.0% to 9.2% with approximately half having A1c values less than the goal of 7.0%. Half the patients were aware of PHRs, majority had smartphones, and all participants had access to the Internet and used email. Two of the patients were retired engineers who had prior experience with software design. The other sessions had between 7 and 8 participants at each session, and 7 patients completed the 90-minute usability testing session. There was a core group of 7 patients who were engaged in the design and testing sessions throughout the entire 9-month study. Key features of the PHR that emerged from design sessions included the following: (1) allow for annotation of data by patients (particularly important for lab values like glucose or for physical activity); (2) calendars, to do list, and reminder functions should be linked so that an entry in one of these allows for auto-population of this data within the other sections; (3) notifications whenever new data from the electronic health record or other sources are pushed to the PHR account; (4) allow for drag and drop of photos of pills/medications taken via smartphone or from other sources so that medication list has photo of actual pills or pill bottle; (5) allow for patients to customize the order of sections in the PHR dashboard so that the sections most important to the individual patient can be displayed more prominently; (6) allow for notifications from pharmacies to be pushed to the PHR (eg, confirmation of receipt of prescription requests or alert that prescription is ready to pick up); and (7) graphical display of trends over time (patients would like to select the measures and time frames to plot for display). Patients cited the importance of data provenance so that patient-entered data Versus provider or electronic health record data could be easily differentiated. Patients also highlighted the importance of having this PHR be a “one-stop shop for all their health data” and to have meaningful data dashboards for the different types of information needed to comprehensively manage their health. Patients wished for a single PHR that could easily bring together data from multiple patient portal accounts to avoid having to manage multiple accounts and passwords. They felt that heat map displays such as those used on popular fitness tracking websites were not intuitive and that the color-coding made interpretation challenging. Participants noted that engagement in the design process made them feel that they contributed towards developing software that could not only positively impact them individually but others as well. Every patient indicated the desire to participate on future design projects. Of the 19 tasks evaluated during usability testing, only 5 tasks could not be completed (eg, adding exercise to the calendar, opening the heat map, etc.). Patients felt that the overall PHR design was clean and aesthetically pleasing. Most patients felt that the site was “pretty easy to use” (6 out of 7). The majority of participants would like to use this PHR in the future (5) and would recommend this PHR to their friends/family to use (6). DISCUSSION/SIGNIFICANCE OF IMPACT: Involving patients directly in the design process for creating a patient-centric connected PHR was essential to sustaining engagement throughout the software life cycle and to informing the design of features and functionalities desired by patients with chronic conditions.

